# Genome-wide characterization of 2-oxoglutarate and Fe(II)-dependent dioxygenase family genes in tomato during growth cycle and their roles in metabolism

**DOI:** 10.1186/s12864-021-07434-3

**Published:** 2021-02-18

**Authors:** Shuo Wei, Wen Zhang, Rao Fu, Yang Zhang

**Affiliations:** Key Laboratory of Bio-resource and Eco-environment of Ministry of Education, College of Life Sciences, Sichuan University, No.24 South Section 1, Yihuan Road, Chengdu, China

**Keywords:** Tomato, 2-Oxoglutarate and Fe(II)-dependent dioxygenase family, Phylogenetics, Expression profile, Metabolism, Growth cycle, Flavone synthase

## Abstract

**Background:**

2-Oxoglutarate and Fe(II)-dependent dioxygenases (2ODDs) belong to the 2-oxoglutarate-dependent dioxygenase (2OGD) superfamily and are involved in various vital metabolic pathways of plants at different developmental stages. These proteins have been extensively investigated in multiple model organisms. However, these enzymes have not been systematically analyzed in tomato. In addition, type I flavone synthase (FNSI) belongs to the 2ODD family and contributes to the biosynthesis of flavones, but this protein has not been characterized in tomato.

**Results:**

A total of 131 2ODDs from tomato were identified and divided into seven clades by phylogenetic classification. The *Sl2ODD*s in the same clade showed similar intron/exon distributions and conserved motifs. The *Sl2ODD*s were unevenly distributed across the 12 chromosomes, with different expression patterns among major tissues and at different developmental stages of the tomato growth cycle. We characterized several *Sl2ODDs* and their expression patterns involved in various metabolic pathways, such as gibberellin biosynthesis and catabolism, ethylene biosynthesis, steroidal glycoalkaloid biosynthesis, and flavonoid metabolism. We found that the *Sl2ODD* expression patterns were consistent with their functions during the tomato growth cycle*.* These results indicated the significance of *Sl2ODDs* in tomato growth and metabolism. Based on this genome-wide analysis of *Sl2ODDs*, we screened six potential *FNSI* genes using a phylogenetic tree and coexpression analysis. However, none of them exhibited FNSI activity.

**Conclusions:**

Our study provided a comprehensive understanding of the tomato 2ODD family and demonstrated the significant roles of these family members in plant metabolism. We also suggest that no *FNSI* genes in tomato contribute to the biosynthesis of flavones.

**Supplementary Information:**

The online version contains supplementary material available at 10.1186/s12864-021-07434-3.

## Background

2-Oxoglutarate-dependent dioxygenases (2OGDs) are soluble, nonheme iron-containing enzymes and constitute the second-largest enzyme family in plants; these enzymes have a highly conserved but not ubiquitous HX(D/E) XnH triad motif in their 2OG-FeII_Oxy (PF03171) domain [1]. The amino acid sequences of plant 2OGD members are highly divergent and can be divided into different types. Analysis of the genomes of six model plant species showed that more than 500 putative 2OGDs could be classified into three major classes: DOXAs, DOXBs and DOXCs [2]. DOXA class enzymes, including plant homologs of *Escherichia coli* (*E.coli*) AlkB, are involved in the oxidative demethylation of alkylated nucleic acids and histones [3]. Prolyl 4-hydroxylase homologs belonging to the DOXB class are involved in proline 4-hydroxylation in cell wall synthesis [4]. Unlike DOXA and B enzymes, which are limited to basic cell functions, DOXC enzymes largely participate in plant primary and secondary metabolism. The functionally characterized DOXC enzymes are involved in several conserved pathways, including hormone metabolism and specific pathways leading to the production of steroidal glycoalkaloids and flavonoids [1]. 2-Oxoglutarate and Fe(II)-dependent dioxygenases (2ODDs) constitute the specific DOXC subfamily and are involved in specialized plant metabolism [5]. In addition to having the classic 2OG-FeII_Oxy (PF03171) domain, they also have the conserved DIOX_N(PF14226) domain [2].

Plants can synthesize massive amount of metabolites due to the diverse biosynthesis-related genes that encode different enzymes [6]. 2ODDs participate in various important metabolic pathways and directly affect the growth, development, and stress responses of plants. Several 2ODDs have been reported to be involved in melatonin metabolism and subsequently affect plant responses to cold, heat, salt, drought, and heavy metal stress and to pathogen invasion [7, 8]. With respect to important plant hormones, such as auxin, ethylene, gibberellin, and salicylic acid, 2ODDs participate in pathways involving their biosynthesis and metabolism [1]. 2ODDs are also involved in the biosynthesis of secondary metabolites that have substantial biological and medicinal value. One 2ODD was identified to promote the biosynthesis of glucoraphasatin in radish [9]. Moreover, a genome-wide study of *Salvia miltiorrhiza* found that 2ODD plays a crucial role in the biosynthesis of tanshinones [10], and 2ODDs in tobacco (*Nicotiana tabacum*) have been functionally characterized as being involved in the biosynthesis of colorful flavonoids [11].

With more than 10,000 known structures, flavonoids are important secondary metabolites [12]. The diverse biological functions of flavonoids in plants as well as their various roles in interactions with other organisms offer many potential applications, from plant breeding to ecology, agriculture, and health benefits for humans [13, 14]. The biosynthesis pathway of flavonoids in the *Solanaceae* has been extensively studied [15, 16]. However, the crucial flavone synthase (FNS) enzymes have not been identified. To date, there are two types of enzymes known to catalyze flavone synthesis in higher plants [17]: FNSIs, a group of soluble 2ODDs, are mainly present in the *Apiaceae* [18], and FNSIIs, a group of NADPH- and molecular oxygen-dependent membrane-bound CYP monooxygenases, are widely distributed across the plant kingdom [19, 20]. OsFNSI was identified using parsley FNSI as bait and is the first FNSI found outside of the *Apiaceae* family [21]. A putative ZmFNSI (*Zea mays*) enzyme has subsequently been found [22]. In addition, the *Arabidopsis* homolog of ZmFNSI also exhibits FNS activity [22]. FNSI is present not only in higher plants but also in liverworts. An FNSI has also been isolated and characterized from *Plagiochasma appendiculatum* [23]. In summary, FNSI is no longer confined to the *Apiaceae* family.

Tomato (*Solanum lycopersicum*), whose fruits are among the most popular fruits worldwide, has become an important source of micronutrients for the human diet and is widely cultivated around the world. Tomato fruits are consumed fresh or as processed products, such as canned tomatoes, paste, puree, ketchup, and juice. In addition to the commercial value of tomato, this species has been studied as a model plant due to its short life cycle and self-compatibility. Tomato plants produce many important primary and secondary metabolites, which can serve as intermediates or substrates for producing valuable new compounds. These advantages make tomato an excellent choice for metabolic engineering to produce important metabolites [24, 25].

A comprehensive analysis of the 2ODD family in tomato has not been performed. In our current study, the Sl2ODDs that belong to the DOXC class were systematically analyzed for their phylogenetic evolution, gene structure, conserved motifs, chromosome location, gene duplications and metabolic pathway involvement. In addition, we verified the potential function of SlFNSI in flavonoid metabolism. Our results offer new insight into the function of 2ODDs in tomato and establish a knowledge base for further genetic improvement of tomato.

## Results and discussion

### Genome-wide identification and phylogenetic analysis of *2ODDs* in tomato

**Fig. 1:**
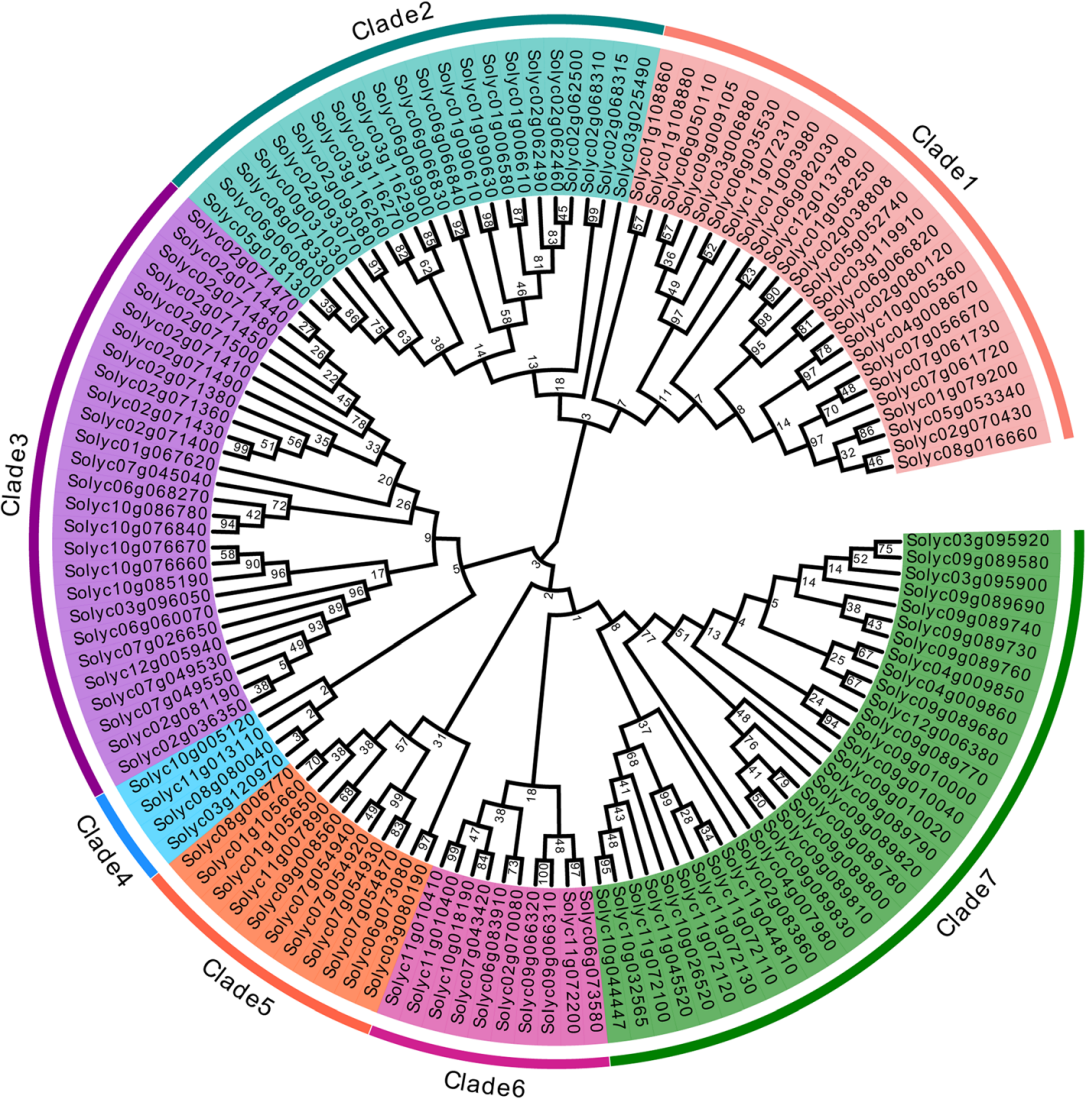
Phylogenetic analysis of tomato 2ODDs. Sl2ODD protein sequences were aligned using MEGA7.0 and evolutionary relationships were determined using Neighbor-Joining tree analysis with 1000 bootstrap replicates. Sl2ODDs fell in seven separate subfamilies named as clade 1-7 and each clade was colored

To investigate 2ODDs involved in plant metabolism, we focused our research on the DOXC subfamily of 2ODDs. A total of 131 putative tomato 2ODDs were found using BLAST and verified using HMMER searches. They all contained two conserved domains, 2OG-FeII_Oxy and DIOX_N. The number of amino acid residues of the predicted Sl2ODDs ranged from 248 to 418, with corresponding molecular weights from 28.4 to 47.7 kDa (Table S1). A phylogenetic tree was constructed to determine the relationships among these Sl2ODDs. The Sl2ODDs could be divided into seven clades (1–7) (Fig. 1). Clade 7 was the largest clade, with 32 members of Sl2ODDs, followed by clade 3, with 27 members. There were 25, 22, 11, and 10 members in clade 1, clade 2, clade 5, and clade 6, respectively. Clade 4 was the smallest, with only four Sl2ODD members. All reported tomato gibberellin oxidases (GAOXs) belonged to clade 1 [26–28]. In addition, 1-aminocyclopropane-1-carboxylic acid oxidases (ACOs) that involved in ethylene biosynthesis were enriched in clade 3 [29]. Taken together, these results showed that our method for retrieving Sl2ODDs is reliable and that our phylogenetic analysis was accurate enough for used in the estimation of the function of several unknown genes. For instance, twenty of the 25 members in clade 1 are GAOXs (Fig. 1), indicating that the remaining five members may also present GAOX activity.

### Gene structure and protein motif analysis of *Sl2ODDs*

**Fig. 2:**
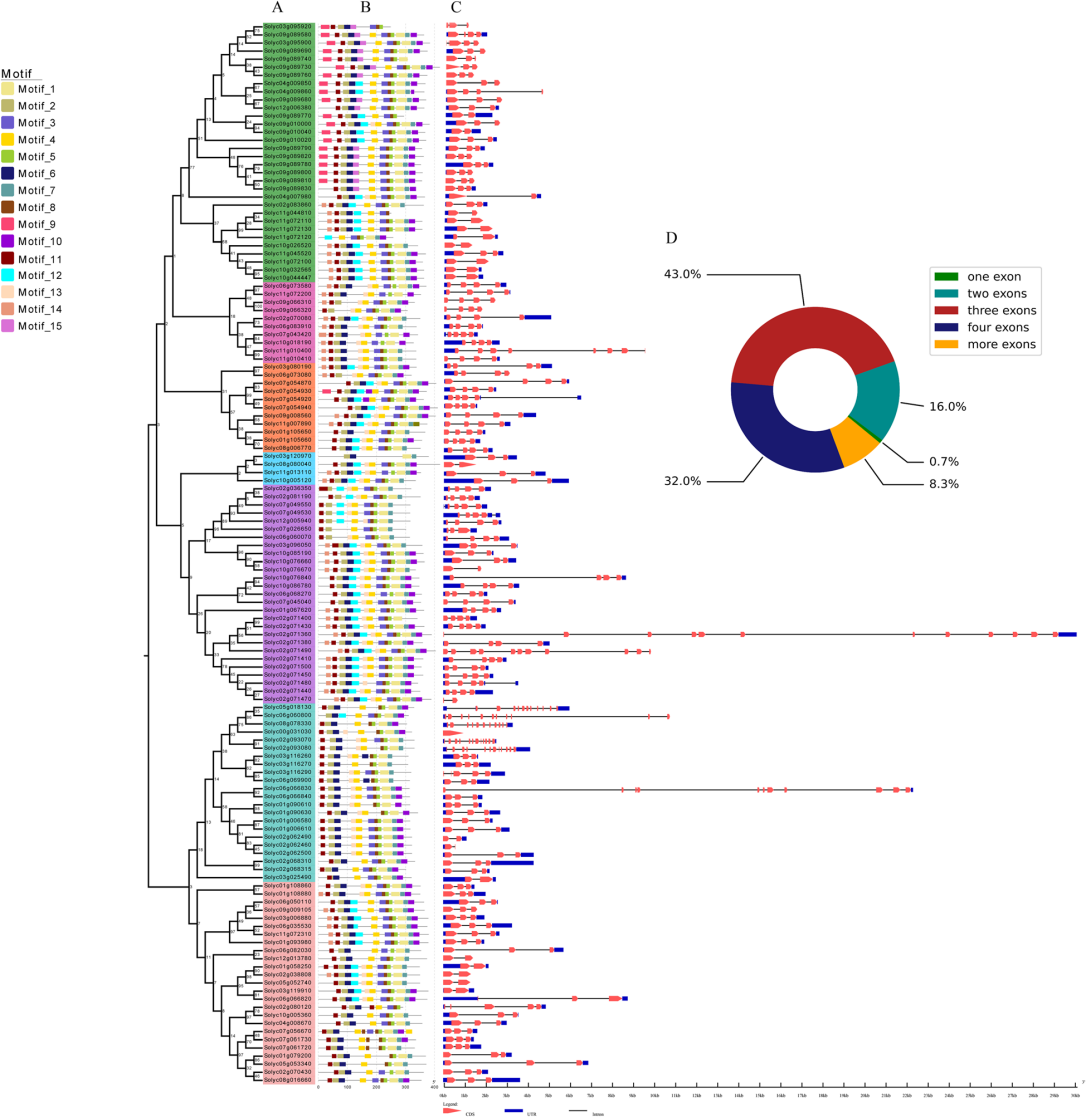
Motif compositions of Sl2ODD proteins and gene structures of *Sl2ODDs* in accordance with the phylogenetic relationships. **a** Phylogenetic relationships of Sl2ODD proteins. **b** Conserved motifs of Sl2ODDs. Each motif is represented in the colored box: motif 1 (khaki), motif 2 (dark khaki), motif 3 (slate blue), motif 4 (gold), motif 5 (yellow green), motif 6 (midnight blue), motif 7 (cadet blue), motif 8 (saddle brown), motif 9 (deep pink), motif 10 (dark violet), motif 11 (dark red), motif 12 (cyan), motif 13 (peach puff), motif 14 (dark salmon), and motif 15 (orchid). **c** Exon and intron gene structures of *Sl2ODDs*. The introns, CDS and UTR are represented by black lines, red wedges, and blue rectangle, respectively. **d** The exon number distributions of *Sl2ODDs*

To gain further insight into the structural diversity of tomato *2ODDs*, we used the online software GSDS 2.0 to analyze the exon-intron structure of *2ODDs* based on the genome sequence and the corresponding coding DNA sequences of the *2ODDs* in tomato (Fig. 2c). The *Sl2ODDs* had 1 ~ 12 exons and could be divided into five categories based on exon number (Fig. 2d). Only *Solyc00g031030* (0.7%) contained one exon. Twenty-two (16%), fifty-five (43%), and forty-two (32%) *Sl2ODDs* contained two, three and four exons, respectively. Eleven (8.3%) members had more than five exons. Notably, the genes from the same clade displayed similar exon numbers (Fig. 2). We identified 15 conserved motifs (1–15) using the online software MEME (Fig. 2b). Motifs 1–8 and 10–11 were widely distributed. Moreover, motifs 9, 12, 13, 14 and 15 were specifically distributed in different clades. The Sl2ODDs within the same clade were found to have similar motif compositions. Overall, the conserved motif composition and gene structure of the 2ODD members, together with the phylogenetic tree results, strongly supported the classification reliability.

### Chromosomal distribution and synteny analysis of *Sl2ODDs*

**Fig. 3:**
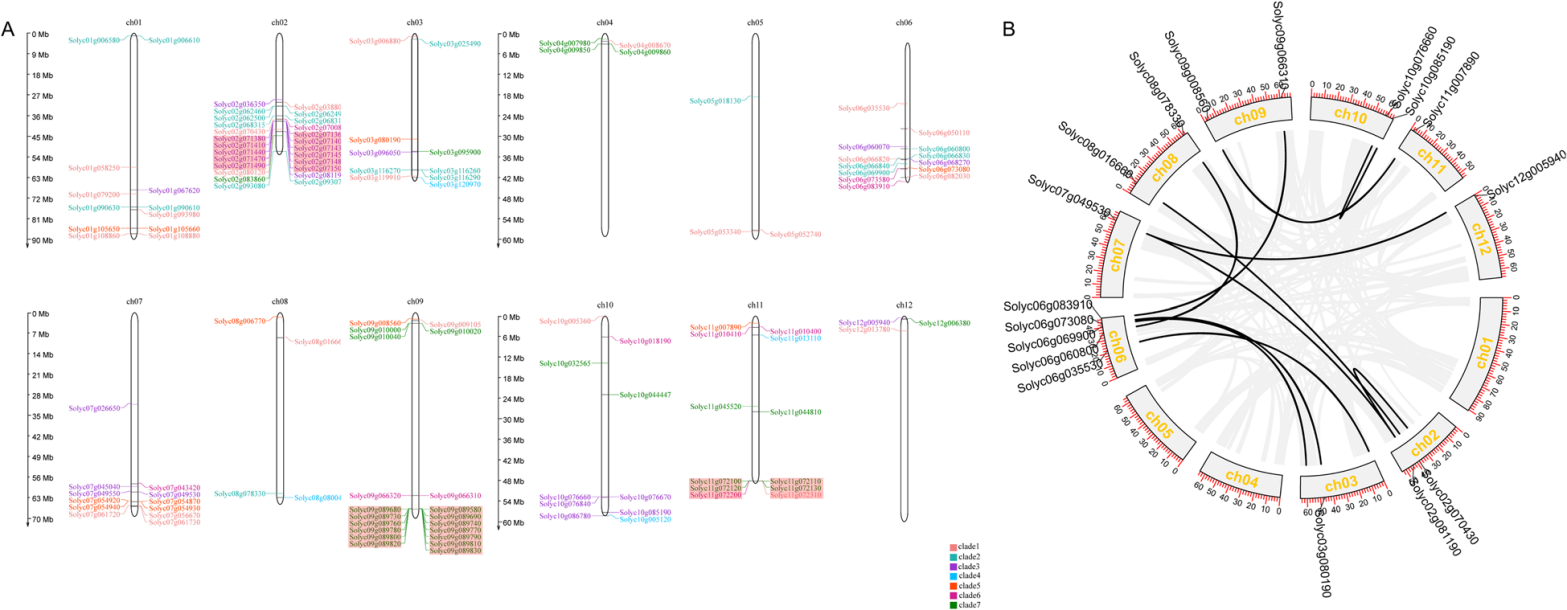
Schematic representations for the distribution and duplication of *Sl2ODD* genes in the tomato genome. **a** The distribution of *Sl2ODDs* in chromosomes. The scale at the left side of figure is shown in Mb. The location of *Sl2ODDs* is indicated on both sides of each chromosome. Different colors of *Sl2ODD*s indicate their subfamilies shown in the Fig.1. **b** The interchromosomal relationships of *Sl2ODDs*. Gray lines indicate all synteny blocks in the tomato genome and the black lines indicate duplicated *Sl2ODD* gene pairs

The 128 *Sl2ODD* members (excluding *Solyc00g031030*, *Solyc10g026520*, and *Solyc03g095920*, which are identified using the MicroTom Metabolic Network (MMN) dataset based on ITAG 3.0 but absent in the updated ITAG 4.0 gene models) are widely distributed across the 12 tomato chromosomes. Chromosome 2 has the largest number of *Sl2ODDs* (25/128). Chromosome 5 and chromosome 11 contain only three *Sl2ODDs*. Most *Sl2ODDs* are located at the proximate or distal end of chromosomes (Fig. 3a). During the progress of plant evolution, gene duplication events contribute significantly to the generation and expansion of gene families. Gene duplication events were also identified for *Sl2ODDs*. We detected duplicated genes in the *Sl2ODD* family using the MCScanX package. Fifty-four (42%) *Sl2ODDs* were confirmed to be tandemly duplicated genes (Fig. S1). We calculated the ka/ks ratios for all tandem genes that were almost less than one, indicating that purifying selection was the main force for 2ODD family gene evolution in tomato (Table S2). According to previously defined criteria [30], a chromosomal region within 200 kb containing two or more genes is defined as the tandem duplication event. Based on the physical location, gene clusters were found on chromosomes 2, 9 and 11 (Fig. 3a), which indicated that tandem gene duplication events happened. However, no further specific functions of these genes were determined. In addition, elven pairs of *Sl2ODDs* were found to be segmental duplicates with the MCScanX method (Fig. 3b). Overall, these results indicated that some *Sl2ODD*s were possibly generated by tandem duplication and segmental duplication events.

### Expression pattern of *Sl2ODDs*

**Fig. 4:**
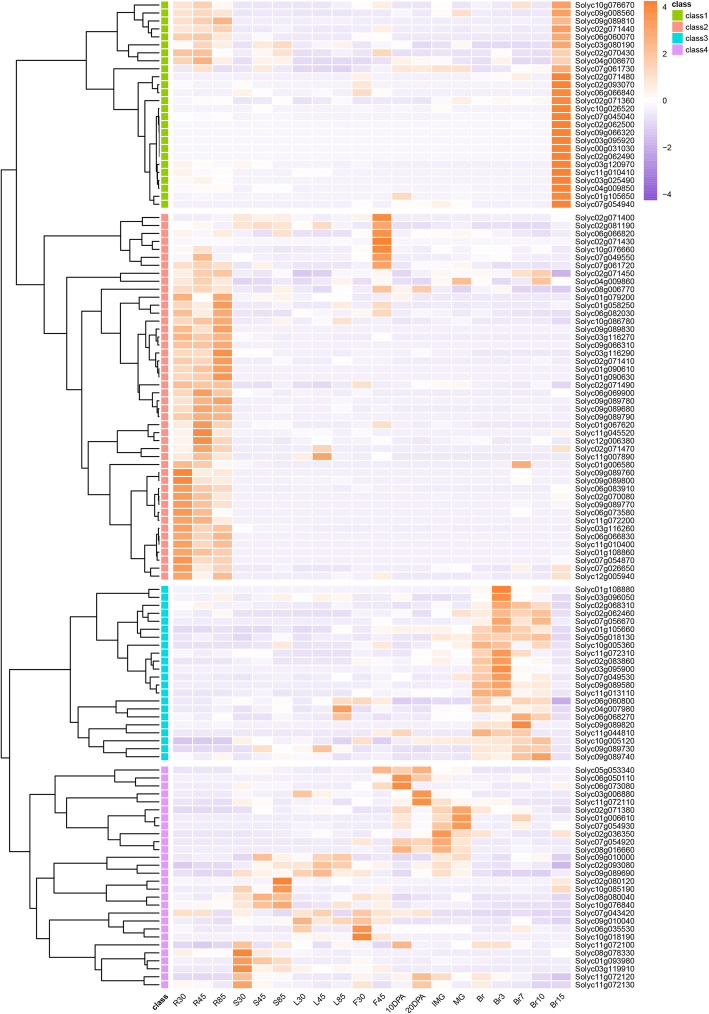
Expression patterns of *Sl2ODDs* during major tomato growth stages and tissues. Data was achieved from MicroTom Metabolic Network (MMN) dataset (Li et al., 2020). X-axis: mRNA levels in 20 different tissues and life stages of MicroTom. R: root, S: stem, L: leaf, F: flower. 30,45,85: days after germination. DPA: days post-anthesis, IMG: immature green, MG: mature green, Br: breaker, Br 3,7,10,15: breaker plus 3,7,10,15 days. Y-axis: Initial of each putative *Sl2ODDs*. Class 1-4 represent four different expression patterns with different colors. Different mRNA levels of each putative *Sl2ODDs* are given as color codes. Purple indicates a low expression level and orange indicates a high expression level

To dissect the potential roles of Sl2ODDs involved in specific plant secondary metabolism, the expression patterns of *Sl2ODD* genes were investigated using the recently published MMN dataset [25]. Seven genes (*Solyc02g038808*, *Solyc02g068315*, *Solyc02g071500*, *Solyc09g009105*, *Solyc09g010020*, *Solyc10g032565 and Solyc10g044447*) were not found in the MMN, and two genes (*Solyc05g052740* and *Solyc12g013780*) were not expressed. The expression patterns of the remaining 122 *Sl2ODDs* could be divided into four clusters (Fig. 4). The most obvious cluster contained 26 *Sl2ODDs* specifically expressed in mature fruit (Br15), including *Solyc09g008560* and *Solyc06g060070* which encode ACOs involved in ethylene biosynthesis. A total of 46 *Sl2ODDs* were mainly expressed in the flowering stage (F45) and the roots. Among them, *SlANS* (*anthocyanidin synthase*) (*Solyc10g076660*) exhibited abundant expression at F45 and was responsible for the synthesis of anthocyanins contributing to the color formation of flowers [31]. Twenty-two *Sl2ODDs* showed high expression levels during fruit development after the breaker (Br) stage, which is the key stage of fruit ripening. *E8* (*Solyc09g089580*), a fruit-specific gene, was a member exhibiting this expression pattern. The last 28 *Sl2ODDs* did not show a particularly consistent expression trend. Interestingly, the expression patterns of some *Sl2ODDs* within the same clade were similar; for example, nearly half of the clade 3 genes (13/27) were expressed significantly in the roots. Similar phenomena occurred for each expression pattern, suggesting a correlation between gene homology and function.

### Potential roles of *Sl2ODDs* in metabolism

2ODDs have been reported to facilitate numerous oxidation reactions such as hydroxylation, halogenation, desaturation, epimerization, cyclization and ring formation, ring cleavage, rearrangement, and demethylation [5, 32]. The impressive versatility of 2ODDs highlights their importance in normal organismal function and has led to high-value specialized metabolites. To describe their potential roles in biosynthesis pathways, the key Sl2ODDs involved in metabolic pathways were analyzed in detail.

### Gibberellin biosynthesis and catabolism

**Fig. 5.**
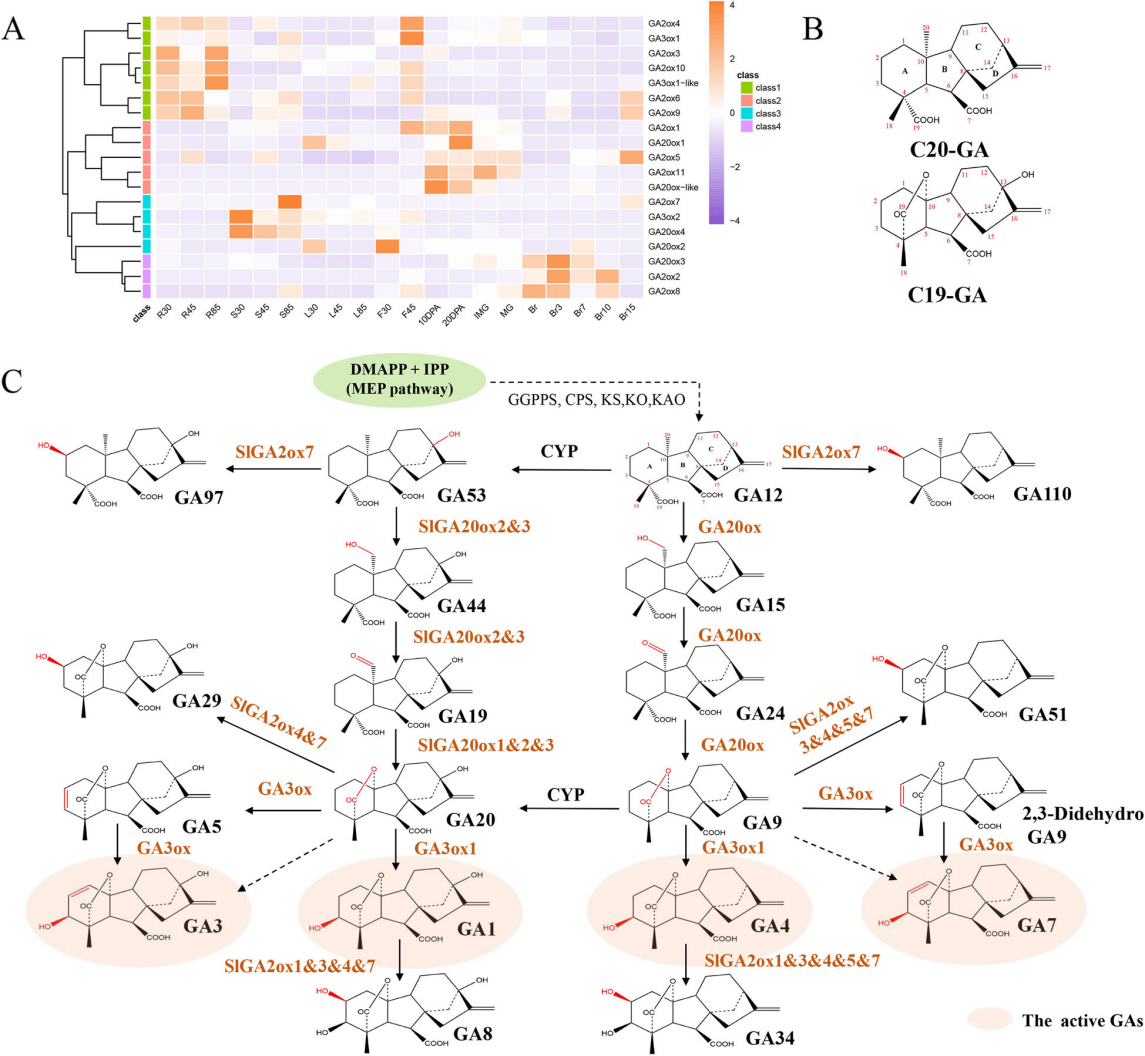
Analysis of *Sl2ODDs* involved in gibberellin biosynthesis and catabolism pathway. **a** Expression profiles of 19 *GAox* genes during the tomato life cycle. **b** Two types of substrate structures for *GAoxs*. **c** The schematic representation of gibberellin biosynthesis and catabolism pathway. Elliptical boxes show active GAs

The plant hormones gibberellins (GA) regulate many plant development stages, including seed germination, cell and shoot elongation, leaf expansion, the transition to flowering, flower growth, and fruit development [33]. In this study, combined with data from published reports [2, 26, 28, 34], we summarized and mapped the gibberellin synthesis and metabolic pathways (Fig. 5c). The well-defined GA biosynthesis and catabolism pathways include three types of GAOXs (GA20OXs, GA3OXs, GA2OXs) that belong to the 2ODD family and contribute to structural modification. GA biosynthesis can occur through two parallel pathways: non-13-hydroxylation and 13-hydroxylation. Carbon-19 (C^− 19^) and carbon-20 (C-20) GAs are two types of substates for GAOXs (Fig. 5b). GA20OXs catalyze the successive oxidation and decarboxylation of C-20 GAs (GA12, GA53) at the C-20 position to form C-19 GAs (GA9, GA20). GA3OXs catalyze the hydroxylation of GA9 and GA20 at the C-3 position to form bioactive GA4 and GA1, respectively. GA2OXs play a role in GA catabolism responsible for GA deactivation via C-2 hydroxylation of the GA backbone. In the present study, a total of 19 putative GAOX coding genes, including 5 *GA20OXs*, 3 *GA3OXs*, and 11 *GA2OXs*, were found in the tomato genome (Fig. 5c).

The expression of *SlGAOXs* had obvious tissue specificity corresponding to significant expression in the roots, stems, flowers, and fruits (Fig. 5a). *GA20OX1–3* and *GA3OX1–2* expression levels have been previously reported [35] and are essentially consistent with our results. In particular, *GA20OX2* was highly expressed in flower buds (F30), and *GA3OX1* was highly expressed at F45 (when 50% of flowers reached anthesis) indicating that the expression of all of them is highly regulated during flower development [35]. Eleven *GA2OXs* were found in tomato, and their expression patterns differed among the different developmental stages and tissues. Overexpression of *GA2OX1* resulted in the reduction in endogenous GAs and led to a decrease in tomato germination rate and fruit weight [26]. The expression patterns of *GA2OX5* and *GA2OX11* are similar to that of *GA2OX1*, and they may jointly regulate the development of tomato fruits and seeds. As GAs have a broad impact on plant growth, according to the expression profile, the different GA2OX homologs in tomato may function in different tissues and periods of plants.

### Ethylene biosynthesis

**Fig. 6:**
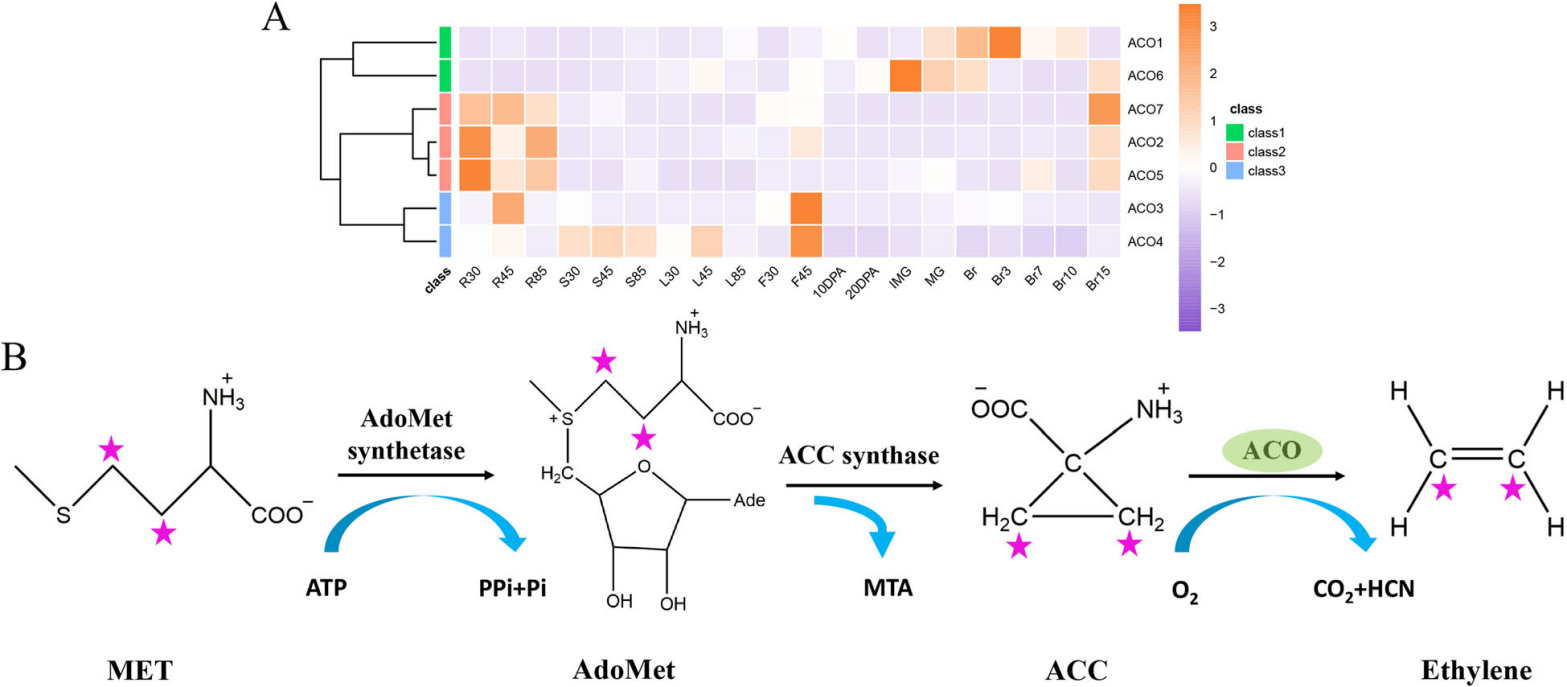
Characterization of *Sl2ODDs* involved in ethylene biosynthesis pathway. **a** Expression profiles of 7 *ACO* genes throughout the tomato life cycle. **b** Schematic representation of ethylene biosynthesis pathway. MET: methionine, AdoMet: S-adenoysl-methionine, ACC: 1-aminocyclopropane-1-carboxylic acid

Ethylene output by organs increases dramatically at specific stages of the plant growth cycle, such as fertilization, ripening, senescence, abscission, and response to stresses [36]. To determine the effect of 2ODDs on ethylene biosynthesis, we mapped the ethylene synthesis pathway (Fig. 6b). Ethylene is derived from the amino acid methionine (MET), catalyzed by AdoMet synthetase and 1-aminocyclopropane-1-carboxylic acid (ACC) synthase, to provide ACC precursors. ACC is then converted into ethylene by ACO, a member of the 2ODD family.

The expression model of the seven *ACOs* during tomato fruit development has been reported previously [29]. We further established the expression profile of *ACOs* throughout the growth cycle using MMN data (Fig. 6a). *ACO1* was mainly expressed in the fruits, suggesting the well-known regulatory effect of ethylene on fruit ripening [29]. *ACO3* and *ACO4* regulate petal senescence and are significantly expressed in flowers (F45), as reported previously [37]. The expression patterns of seven *ACOs* were different, indicating that their roles in the plant may be diverse.

### Steroidal glycoalkaloid (SGA) biosynthesis

**Fig. 7:**
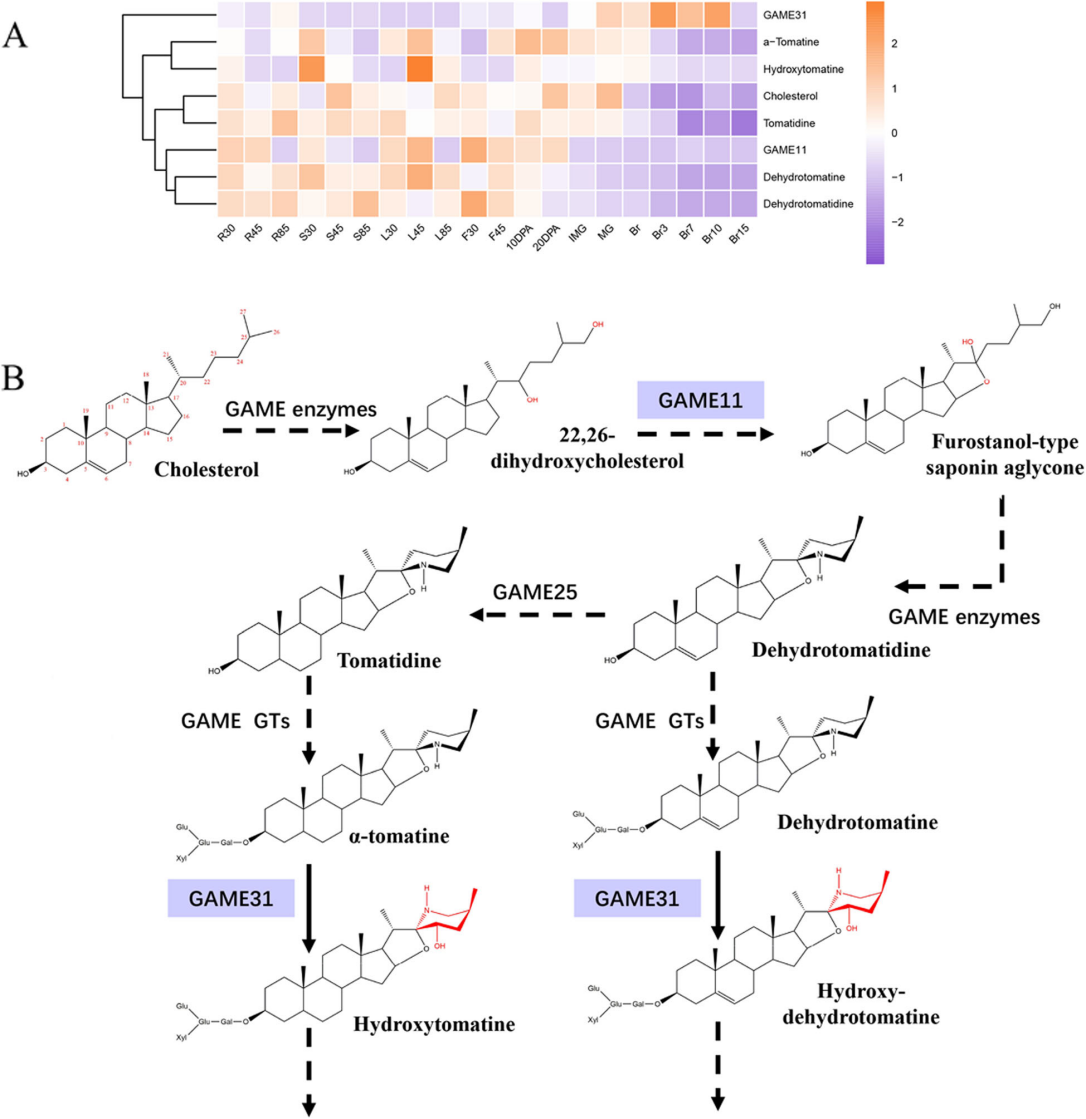
Two *Sl2ODDs* participated in steroidal glycoalkaloids (SGA) biosynthesis pathway. **a** Coexpression analysis of two *GAME* genes belonging to the 2ODD family with major SGAs across the tomato life cycle. Data was extracted from MMN (Li et al., 2020). **b** Schematic representation of SGA biosynthesis pathway. Purple boxes represent Sl2ODDs

Steroidal glycoalkaloids and their derivatives, mainly α-tomatine and dehydrotomatine, are cytotoxic antinutritional compounds and accumulate in immature tomato fruits [38]. Cholesterol is the proposed common precursor for the biosynthesis of SGAs. A series of *GLYCOALKALOID METABOLISM* (*GAME*) genes are responsible for the hydroxylation, oxidation, and transamination of SGAs (Fig. 7b). Two of them, *GAME11* and *GAME31*, are *2ODDs*. *GAME11* participates in the initial synthesis process of SGAs and was highly expressed in the roots, leaves, flowers, and immature green fruits (Fig. 7a). In contrast, *GAME31* was mainly expressed at the tomato fruit ripening stage and catalyzes the first important step in the chemical shift after maturation within nonbitter SGA by a hydroxylation reaction [39, 40]. Interestingly, we found that the different expression patterns between *GAME11* and *GAME31* resulted in the appropriate function at the right time. To gain further insight into the spatiotemporal specificities of compounds in different tissues, the coexpression of metabolites and genes was analyzed (Fig. 7a). The upstream SGA metabolites accumulated mainly in the leaves (L45) and green fruits, which is in line with the expression pattern of the upstream biosynthesis-related gene *GAME11*. The content of downstream SGAs decreases gradually after the Br period along with the expression of the downstream biosynthesis-related gene *GAME31*. These results are consistent with those of a previous study [40].

### Flavonoid biosynthesis and metabolism

**Fig. 8:**
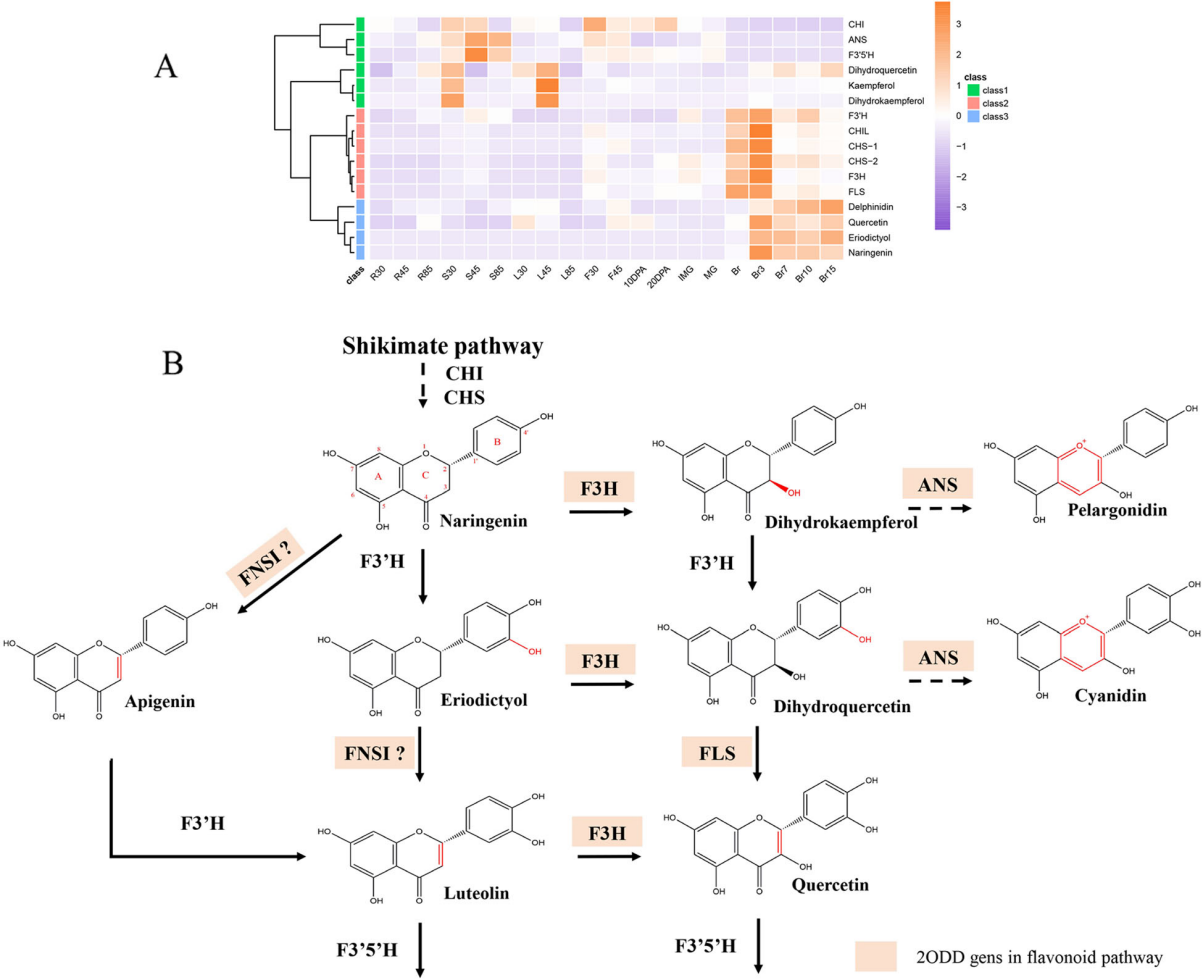
Characterization of *Sl2ODDs* in flavonoids biosynthesis pathway. **a** Coexpression analysis of genes and flavonoids across the tomato life cycle. Data was achieved from MMN (Li et al., 2020). **b** Schematic representation of flavonoids biosynthesis pathway. Square boxes show 2ODDs in flavonoids pathway. CHI: chalcone isomerase, CHS: chalcone synthase, F3H: flavanone-3-hydroxylase, F3’H: flavonoid 3’-hydroxylase, FLS: flavonol synthase, ANS: anthocyanidin synthase, F3’5’H: flavonoid 3’,5’-hydroxylase. So far, there is no evidence of the presence of FNSI (Flavone synthase I) in tomato

As shown in Fig. 8b, flavonoids are derived from the shikimate pathway, and the committed steps are catalyzed by chalcone synthase (CHS) and chalcone isomerase (CHI) to yield naringenin, which is subsequently modified by different enzymes, including cytochrome P450s (CYPs) and 2ODDs. FNSI, F3H, flavonol synthase (FLS), and anthocyanidin synthase (ANS) are flavonoid dioxygenases and belong to the 2ODD family. Based on the MMN data, we conducted a coexpression analysis of genes and compounds of the flavonoid pathway. *Flavonoid 3′-hydroxylase* (*F3’H*), *CHI-like* (*CHIL*), *CHS-1*, *CHS-2*, *F3H*, and *FLS* exhibited similar expression patterns during the tomato growth cycle. The accumulation of their corresponding products, such as eriodictyol and quercetin, followed (Fig. 8a). These results suggested that performing a coexpression analysis might be a reliable approach to study gene function.

**Fig. 9:**
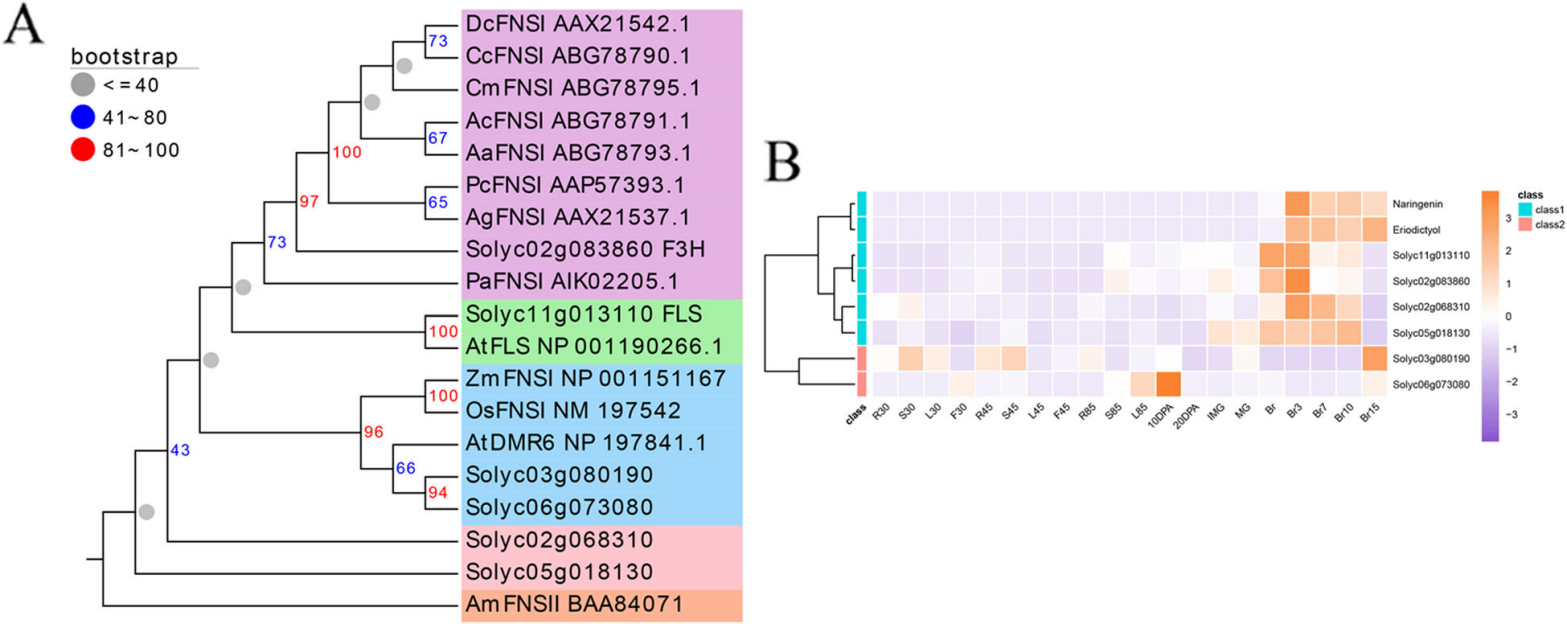
Selection of potential *SlFNSIs*. **a** Phylogenetic analysis of potential SlFNSIs and other known FNSIs. **b** Coexpression analysis of potential *SlFNSIs* and flavonoids across the tomato life cycle. Data was obtained from MMN dataset (Li et al.,2020)

Although the flavonoid pathway of plants has been studied [15, 16], the key enzyme FNS responsible for the production of flavones, which compose the largest subgroup of flavonoids, has not been reported in the *Solanaceae* thus far. Studies have showed conflicting results regarding the presence of flavones in *Solanaceae* species, including tomato [41–44]. To further determine whether type I flavone synthase (FNSI) exists in tomato, a total of six candidate genes (*Solyc02g068310*, *Solyc05g018130*, *Solyc03g080190*, *Solyc06g073080*) including both *F3H* (*Solyc02g083860*) and *FLS* (*Solyc11g013110*), were selected based on phylogenetic tree analysis and coexpression analysis (Fig. 9). Two candidates (*Solyc03g080190* and *Solyc06g073080*) along with other FNSIs (ZmFNSI, OsFNSI, AtDMR6) were distributed in the same group (blue). The other four candidates, *Solyc02g068310*, *Solyc05g018130*, *F3H*, and *FLS* exhibited coexpression patterns together with those of the accumulation of upstream compounds (Fig. 9b). The expression of these six potential genes may lead to FNS activity in tomato.

**Fig. 10:**
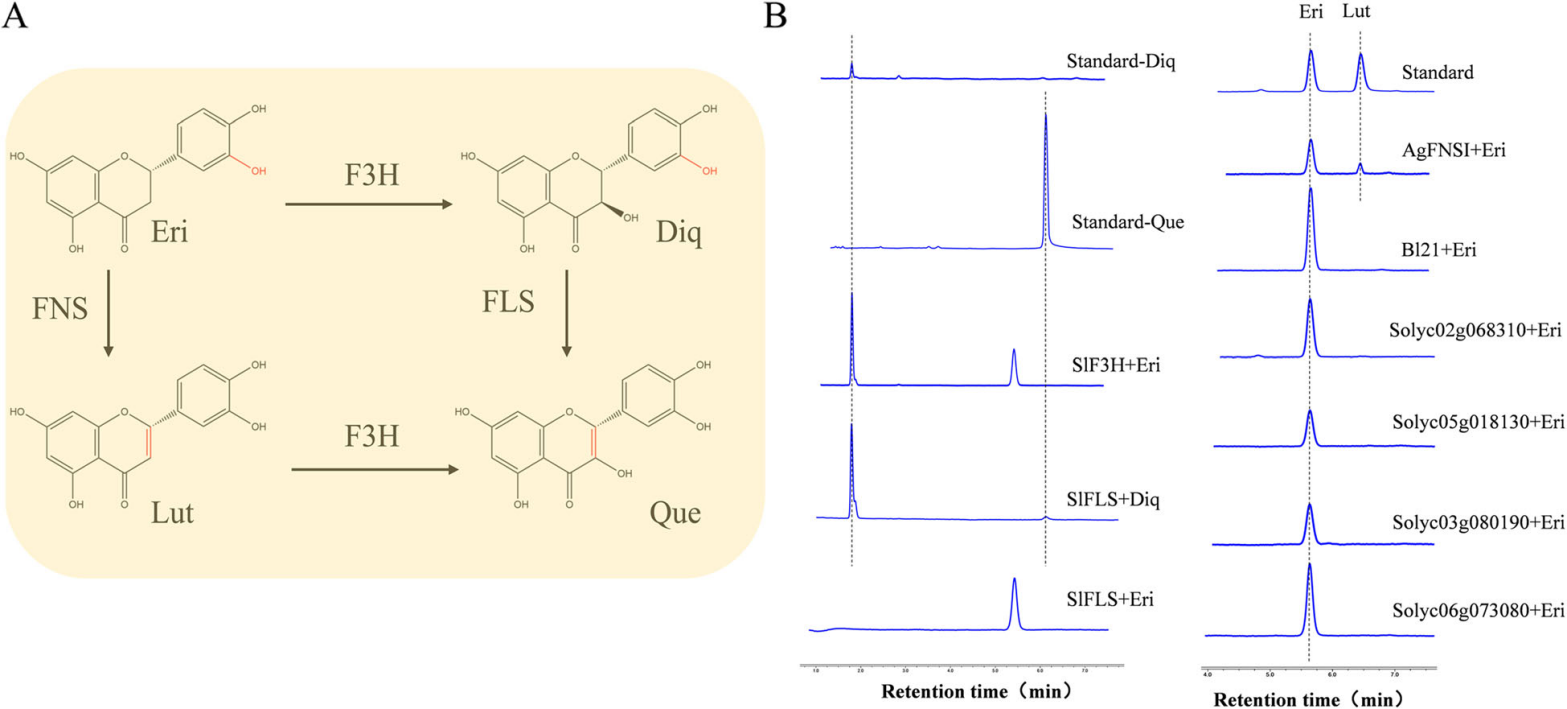
No FNSI activity was confirmed using candidate Sl2ODDs. **a** Schematic diagram of the reactions of eriodictyol under the action of different enzymes. Eri: eriodictyol, Diq: dihydroquercetin, Lut: luteolin, Que: quercetin. FNS: Flavone synthase, F3H: flavanone-3-hydroxylase, FLS: flavonol synthase. **b** UPLC analysis of *in vitro* enzyme reaction products of SlFNSIs. SlFLS-Diq/Eri: SlFLS uses Diq and Eri as substrates, respectively. The remaining enzymes use Eri as a substrate to verify the FNS activity

We cloned and expressed these candidates to test their FNS ability by converting flavanone (eriodictyol, Eri) into the corresponding flavone (luteolin, Lut) (Fig. 10a). *AgFNSI* (*Apium graveolens*) was used as a positive control [18] and showed FNS activity. F3H converted Eri into the flavanonol product dihydroquercetin (Diq), and FLS converted Diq into quercetin (Que), as expected. However, these candidates did not show FNS activities (Fig. 10a and b). Regarding the other candidate genes, none of them exhibited FNS activity. All six potential genes failed to present FNS activity.

## Conclusions

In this study, a total of 131 *2ODDs* were identified in the tomato genome, and their phylogenetic relationships, structures, chromosomal locations, duplications, and expression patterns were investigated. We found that the *Sl2ODDs* within the same clades share a similar motif composition and structure, inferring that they may have the same conserved function. The expression profile suggested that *Sl2ODD*s were widely distributed in different tissues and stages, revealing their importance for normal organismal function during the tomato growth cycle. Our results highlighted their irreplaceable roles in the biosynthesis of gibberellins, ethylene, steroidal glycoalkaloids, and flavonoids. Importantly, we characterized six potential *Sl2ODD*s encoding FNSI and concluded that there was no functional FNSI in tomato. Our findings promote the understanding of the evolution and function of *2ODDs* in tomato, and therefore provide a reference for further research, especially for the genetic improvement of the tomato flavonoid pathway.

## Methods

### Plant material and growth conditions

The seeds of *Solanum lycopersicum* cv MicroTom were purchased from PanAmerican Seed (Illinois, USA). The resulting plants were grown in a greenhouse under a 16 h light/8 h dark photoperiod, 24 °C and under 60% humidity [45].

### Retrieval of putative 2ODDs from tomato

We performed a repeated BLASTP search (e values of < 0.001) using the 2OG-FeII_Oxy (PF03171) and DIOX_N(PF14226) domains of tomato flavanone-3-hydroxylase (SlF3H, Solyc02g083860) against the tomato transcriptome (ITAG 4.0) downloaded from the tomato genome database (ftp://ftp.solgenomics.net/tomato_genome/annotation/ITAG4.0_release/) [46] and combined the annotation data of the recently established MicroTom Metabolic Network (MMN, https://www.sciencedirect.com/science/article/pii/S1674205220301830) [25] to identify *2ODD* genes in the *Solanum lycopersicum* genome. All the candidate sequences were further verified by a Hidden Markov Model (HMM) search using PFAM (http://pfam.xfam.org/) [47] and SMART (http://smart.embl-heidelberg.de/) [48].

### Phylogenetic analysis

A total of 131 identified tomato 2ODDs were used for multiple protein sequence alignments via ClustalW in MEGA 7.0 (https://www.megasoftware.net/) [49]. The alignment results were subsequently used to construct a phylogenetic tree using the neighbor-joining method with 1000 bootstrap replicates and complete deletion. The other parameters were set to the defaults. The phylogenetic tree was displayed with the online tool EvolView (https://evolgenius.info//evol-view-v2) [49].

### Gene structure and conserved motif analysis

Gene structures were analyzed based on the full-length genome sequence using the online tool Gene Structure Display Server (GSDS) 2.0 (http://gsds.gao-lab.org/index.php) [50]. To identify conserved motifs, the MEME online website (https://meme.n-bcr.net/meme) [51] was used with the following parameters: maximum number of motifs, 15; optimum width of each motif, between 12 and 30 residues; and optional parameters, default values. The characteristics of the 2ODD structures with motif compositions were visualized by EvolView.

### Chromosomal location and synteny analysis

All *2ODDs* were mapped to the 12 tomato chromosomes based on physical location information from the database of the tomato genome using MG2C 2.1 (http://mg2c.iask.in/mg2c_v2.1/) [52]. Analysis of gene duplications was conducted with MCScanX software (http://chibba.pgml.uga.edu/mcscan2/) [53] using the amino acid sequences and chromosomal positioning data of *2ODDs*, after which the results were visualized using TBtools (https://github.com/CJ-Chen/TBtools) [54]. The nonsynonymous (Ka) and synonymous substitution (Ks) rates of duplicated *2ODD* genes were calculated using KaKs_Calculator 2.0 (http://www.bork.embl.d-e/pal2nal/). The ratio was then calculated to evaluate the selection pressure.

### Expression patterns of *Sl2ODDs*

The MicroTom Metabolic Network (MMN), a high-temporal-resolution transcriptome and metabolome dataset that contains data from 20 different tissues and stages during the MicroTom growth cycle, was used to study the expression patterns of *2ODDs* (https://www.sciencedirect.com/science/article/pii/S1674205220301830) [25]. A heatmap of *2ODD* expression was created and displayed using the R language program. Transcript abundance was calculated as fragments per kilobase of exon model per million mapped reads, and the resulting values were z-score transformed to normalize the gene expression levels.

### Coexpression analysis

Coexpression analysis was conducted for 20 different time points/tissue samples by R software with the heatmap package. The normalized expression values of genes and metabolites were calculated by the z-score method which is a built-in standardized function of R software.

### Clone and expression of potential *SlFNSIs*

The full-length CDSs of potential *SlFNSIs* were amplified using polymerase chain reaction (PCR) from cDNA in conjunction with primers designed based on the sequences obtained from the tomato genome database (https://solgenomics.net/). The CDSs were cloned into a pDONR207, sequenced, and subsequently recombined into pDEST17 vector through Gateway cloning [45]. The potential *SlFNSIs* were expressed in *E.coli* strain BL21 grown at 37 °C in Luria-Bertani (LB) media containing 0.05 mg ml^− 1^ carbenicillin until the optical density at 600 nm reached 0.7–0.9. Recombinant proteins were expressed by induction with 0.5 mM isopropyl β-D-1-thiogalactopyranoside (IPTG) for 18 h at 16 °C. Cells from 40 ml of culture were harvested by centrifugation and resuspended in 3 ml of PBS buffer (pH 7.0) at 4 °C. Afterward, cell lysis was performed using an ultrasonic homogenizer and the lysates were recovered by centrifugation (10,000 g) for 20 min [55].

### In vitro enzyme assays

The potential crude SlFNSI enzymes were incubated together with 160 μM α-oxoglutarate, 50 μM ferrous sulfate, and 200 μM eriodictyol in a final volume of 100 μl of PBS buffer (pH 7.0) for 1 h at 30 °C. The reaction was stopped by the addition of 400 μl of methanol. The mixture was then centrifuged at 20,000 *g* at 4 °C for 10 min after which the supernatant was collected for measurements.

### Ultra-performance liquid chromatography (UPLC) analysis

The product analysis was performed on a Dionex Ultimate 3000 Series UPLC (Thermo Scientific, MA, USA) and a 100 × 2.1 mm 1.9 μm Hypersil Gold C18 column (Thermo Scientific, MA, USA) with 100% acetonitrile used as mobile phase A and 0.1% formic acid in ultrapure water used as mobile phase B, running at 0.5 ml/min and 40 °C. The mobile gradient was as follows: 0–2 min, 83% B; 2–5 min, 83–80% B; 5–7 min, 80–75% B; 7–11 min, 75% B; 11–11.1 min, 75–83% B; and 11.1–13 min, 83% B. Detection was performed at 280 nm for eriodictyol and 350 nm for luteolin.

## Supplementary Information

**Additional file 1: Fig. S1.** Distributions of 54 tandem duplicated *Sl2ODD* genes in the tomato genome. The red lines indicated the duplicated *Sl2ODD* gene pairs (Fig. 3b).

**Additional file 2.**

**Additional file 3.**

## Data Availability

The datasets generated and analyzed during the current study are available in the Sol Genomics Network repository (Tomato Genome version SL4.0 and Annotation ITAG4.0, ftp://ftp.solgenomics.net/tomato_genome/annotation/ITAG4.0_release/). The metabolites content and gene expression data used in this study can be obtained from the MMN dataset (Li et al.,2020) (https://www.sciencedirect.com/science/article/pii/S1674205220301830). The amino acid sequences used for phylogenetic trees were downloaded from the NCBI database. The accession numbers are included in the figures.

## Abbreviations

2ODD: 2-oxoglutarate and Fe(II)-dependent dioxygenases
FNSI: Type I flavone synthase
FNSII: Type II flavone synthase
CYP450: Cytochrome P450
MMN: MicroTom Metabolic Network
GA20OX: Gibberellin 20-oxidase
GA3OX: Gibberellin 3-oxidase
GA2OX: Gibberellin 2-oxidase
ACO: 1-aminocyclopropane-1-carboxylic acid oxidase
GAME: GLYCOALKALOID METABOLISM
SGA: Steroidal glycoalkaloid
CHI: Chalcone isomerase
CHS: Chalcone synthase
F3H: Flavanone 3-hydroxylase
F3’H: Flavonoid 3′-hydroxylase
FLS: Flavonol synthase
ANS: Anthocyanidin synthase
F3’5’H: Flavonoid 3′,5′-hydroxylase
Eri: Eriodictyol
Diq: Dihydroquercetin
Lut: Luteolin
Que: Quercetin
DMR6: Downy mildew resistance 6

## References

[1] Hagel JM, Facchini PJ (2018). Expanding the roles for 2-oxoglutarate-dependent oxygenases in plant metabolism. Nat Prod Rep.

[2] Kawai Y, Ono E, Mizutani M (2014). Evolution and diversity of the 2-oxoglutarate-dependent dioxygenase superfamily in plants. Plant J.

[3] Mielecki D, Zugaj DL, Muszewska A, Piwowarski J, Chojnacka A, Mielecki M, Nieminuszczy J, Grynberg M, Grzesiuk E (2012). Novel AlkB dioxygenases--alternative models for in silico and in vivo studies. PLoS One.

[4] Keskiaho K, Hieta R, Sormunen R, Myllyharju J (2007). Chlamydomonas reinhardtii has multiple prolyl 4-hydroxylases, one of which is essential for proper cell wall assembly. Plant Cell.

[5] Farrow SC, Facchini PJ (2014). Functional diversity of 2-oxoglutarate/Fe (II)-dependent dioxygenases in plant metabolism. Front Plant Sci.

[6] Fu R, Martin C, Zhang Y (2018). Next-generation plant metabolic engineering, inspired by an ancient Chinese irrigation system. Mol Plant.

[7] Lee K, Zawadzka A, Czarnocki Z, Reiter RJ, Back K (2016). Molecular cloning of melatonin 3-hydroxylase and its production of cyclic 3-hydroxymelatonin in rice (Oryza sativa). J Pineal Res.

[8] Yu Y, Lv Y, Shi Y, Li T, Chen Y, Zhao D, Zhao Z (2018). The Role of Phyto-Melatonin and Related Metabolites in Response to Stress. Molecules.

[9] Kakizaki T, Kitashiba H, Zou Z, Li F, Fukino N, Ohara T, Nishio T, Ishida M (2017). A 2-Oxoglutarate-dependent Dioxygenase mediates the biosynthesis of Glucoraphasatin in radish. Plant Physiol.

[10] Xu Z, Song J (2017). The 2-oxoglutarate-dependent dioxygenase superfamily participates in tanshinone production in Salvia miltiorrhiza. J Exp Bot.

[11] Wang Z, Wang S, Wu M, Li Z, Liu P, Li F, Chen Q, Yang A, Yang J (2019). Evolutionary and functional analyses of the 2-oxoglutarate-dependent dioxygenase genes involved in the flavonoid biosynthesis pathway in tobacco. Planta.

[12] Panche AN, Diwan AD, Chandra SR (2016). Flavonoids: an overview. J Nutr Sci.

[13] Madunic J, Madunic IV, Gajski G, Popic J, Garaj-Vrhovac V (2018). Apigenin: a dietary flavonoid with diverse anticancer properties. Cancer Lett.

[14] Imran M, Rauf A, Abu-Izneid T, Nadeem M, Shariati MA, Khan IA, Imran A, Orhan IE, Rizwan M, Atif M (2019). Luteolin, a flavonoid, as an anticancer agent: a review. Biomed Pharmacother.

[15] Tohge T, de Souza LP, Fernie AR (2017). Current understanding of the pathways of flavonoid biosynthesis in model and crop plants. J Exp Bot.

[16] Liu Y, Tikunov Y, Schouten RE, Marcelis LFM, Visser RGF, Bovy A (2018). Anthocyanin biosynthesis and degradation mechanisms in Solanaceous vegetables: a review. Front Chem.

[17] Martens S, Mithofer A (2005). Flavones and flavone synthases. Phytochemistry.

[18] Tan GF, Ma J, Zhang XY, Xu ZS, Xiong AS (2017). AgFNS overexpression increase apigenin and decrease anthocyanins in petioles of transgenic celery. Plant Sci.

[19] Kitada C, Gong Z, Tanaka Y, Yamazaki M, Saito K (2001). Differential expression of two cytochrome P450s involved in the biosynthesis of flavones and anthocyanins in chemo-varietal forms of Perilla frutescens. Plant Cell Physiol.

[20] Zhao Q, Zhang Y, Wang G, Hill L, Weng JK, Chen XY, Xue HW, Martin C (2016). A specialized flavone biosynthetic pathway has evolved in the medicinal plant, Scutellaria baicalensis. Sci Adv.

[21] Lee YJ, Kim JH, Kim BG, Lim Y, Ahn JH. Characterization of flavone synthase I from rice. BMB Rep. 2008; 41(1):68-71. 10.5483/bmbrep.2008.41.1.068 18304453

[22] Falcone Ferreyra ML, Emiliani J, Rodriguez EJ, Campos-Bermudez VA, Grotewold E, Casati P (2015). The identification of maize and Arabidopsis type I flavone Synthases links flavones with hormones and biotic interactions. Plant Physiol.

[23] Han XJ, Wu YF, Gao S, Yu HN, Xu RX, Lou HX, Cheng AX (2014). Functional characterization of a Plagiochasma appendiculatum flavone synthase I showing flavanone 2-hydroxylase activity. FEBS Lett.

[24] Li Y, Wang H, Zhang Y, Martin C (2018). Can the world's favorite fruit, tomato, provide an effective biosynthetic chassis for high-value metabolites?. Plant Cell Rep.

[25] Li Y, Chen Y, Zhou L, You S, Deng H, Chen Y, Alseekh S, Yuan Y, Fu R, Zhang Z (2020). MicroTom metabolic network: rewiring tomato metabolic regulatory network throughout the growth cycle. Mol Plant.

[26] Chen S, Wang X, Zhang L, Lin S, Liu D, Wang Q, Cai S, El-Tanbouly R, Gan L, Wu H (2016). Identification and characterization of tomato gibberellin 2-oxidases (GA2oxs) and effects of fruit-specific SlGA2ox1 overexpression on fruit and seed growth and development. Hortic Res.

[27] Serrani JC, Ruiz-Rivero O, Fos M, Garcia-Martinez JL (2008). Auxin-induced fruit-set in tomato is mediated in part by gibberellins. Plant J.

[28] Garcia-Hurtado N, Carrera E, Ruiz-Rivero O, Lopez-Gresa MP, Hedden P, Gong F, Garcia-Martinez JL (2012). The characterization of transgenic tomato overexpressing gibberellin 20-oxidase reveals induction of parthenocarpic fruit growth, higher yield, and alteration of the gibberellin biosynthetic pathway. J Exp Bot.

[29] Houben M, Van de Poel B (2019). 1-Aminocyclopropane-1-carboxylic acid oxidase (ACO): the enzyme that makes the plant hormone ethylene. Front Plant Sci.

[30] Holub EB (2001). The arms race is ancient history in Arabidopsis, the wildflower. Nat Rev Genet.

[31] Zhang Y, Butelli E, Martin C (2014). Engineering anthocyanin biosynthesis in plants. Curr Opin Plant Biol.

[32] Tarhonskaya H, Szollossi A, Leung IK, Bush JT, Henry L, Chowdhury R, Iqbal A, Claridge TD, Schofield CJ, Flashman E (2014). Studies on deacetoxycephalosporin C synthase support a consensus mechanism for 2-oxoglutarate dependent oxygenases. Biochemistry.

[33] Nagel R (2020). Gibberellin signaling in plants: entry of a new MicroRNA player. Plant Physiol.

[34] Serrani JC, Sanjuan R, Ruiz-Rivero O, Fos M, Garcia-Martinez JL (2007). Gibberellin regulation of fruit set and growth in tomato. Plant Physiol.

[35] Mariken R, Tsuyoshi K, Hiroshi K, Shinjiro Y, Young-Yell Y, Ryozo I, Hiroyuki S, Yuji K (1999). Regulation of gibberellin biosynthesis genes during flower and early fruit development of tomato. Plant J.

[36] Li S, Chen K, Grierson D (2019). A critical evaluation of the role of ethylene and MADS transcription factors in the network controlling fleshy fruit ripening. New Phytol.

[37] Llop-Tous I, Barry CS, Grierson D (2000). Regulation of ethylene biosynthesis in response to pollination in tomato flowers. Plant Physiol.

[38] Milner SE, Brunton NP, Jones PW, O’Brien NM, Collins SG, Maguire AR (2011). Bioactivities of glycoalkaloids and their aglycones from Solanum species. J Agric Food Chem.

[39] Itkin M, Heinig U, Tzfadia O, Bhide AJ, Shinde B, Cardenas PD, Bocobza SE, Unger T, Malitsky S, Finkers R (2013). Biosynthesis of antinutritional alkaloids in solanaceous crops is mediated by clustered genes. Science.

[40] Cardenas PD, Sonawane PD, Heinig U, Jozwiak A, Panda S, Abebie B, Kazachkova Y, Pliner M, Unger T, Wolf D (2019). Pathways to defense metabolites and evading fruit bitterness in genus Solanum evolved through 2-oxoglutarate-dependent dioxygenases. Nat Commun.

[41] Alarcón-Flores MI, Romero-González R, Martínez Vidal JL, Garrido Frenich A (2015). Multiclass determination of phenolic compounds in different varieties of tomato and lettuce by ultra high performance liquid chromatography coupled to tandem mass spectrometry. Int J Food Prop.

[42] Tsugawa H, Nakabayashi R, Mori T, Yamada Y, Takahashi M, Rai A, Sugiyama R, Yamamoto H, Nakaya T, Yamazaki M (2019). A cheminformatics approach to characterize metabolomes in stable-isotope-labeled organisms. Nat Methods.

[43] Zhu GT, Wang SC, Huang ZJ, Zhang SB, Liao QG, Zhang CZ, Lin T, Qin M, Peng M, Yang CK (2018). Rewiring of the Fruit Metabolome in Tomato Breeding. Cell.

[44] Palomo I, Concha-Meyer A, Lutz M, Said M, Saez B, Vasquez A, Fuentes E (2019). Chemical Characterization and Antiplatelet Potential of Bioactive Extract from Tomato Pomace (Byproduct of Tomato Paste). Nutrients.

[45] Ying S, Su M, Wu Y, Zhou L, Fu R, Li Y, Guo H, Luo J, Wang S, Zhang Y (2020). Trichome regulator SlMIXTA-like directly manipulates primary metabolism in tomato fruit. Plant Biotechnol J.

[46] Zhao P, Wang D, Wang R, Kong N, Zhang C, Yang C, Wu W, Ma H, Chen Q (2018). Genome-wide analysis of the potato Hsp20 gene family: identification, genomic organization and expression profiles in response to heat stress. BMC Genomics.

[47] El-Gebali S, Mistry J, Bateman A, Eddy SR, Luciani A, Potter SC, Qureshi M, Richardson LJ, Salazar GA, Smart A (2019). The Pfam protein families database in 2019. Nucleic Acids Res.

[48] Letunic I, Bork P (2018). 20 years of the SMART protein domain annotation resource. Nucleic Acids Res.

[49] Kumar S, Stecher G, Tamura K (2016). MEGA7: molecular evolutionary genetics analysis version 7.0 for bigger datasets. Mol Biol Evol.

[50] Hu B, Jin J, Guo AY, Zhang H, Luo J, Gao G (2015). GSDS 2.0: an upgraded gene feature visualization server. Bioinformatics.

[51] Bailey TL, Boden M, Buske FA, Frith M, Grant CE, Clementi L, Ren J, Li WW, Noble WS (2009). MEME SUITE: tools for motif discovery and searching. Nucleic Acids Res.

[52] Jiangtao C, Yingzhen K, Qian W, Yuhe S, Daping G, Jing L, Guanshan L (2015). MapGene2Chrom, a tool to draw gene physical map based on Perl and SVG languages. Yi Chuan.

[53] Wang Y, Tang H, Debarry JD, Tan X, Li J, Wang X, Lee TH, Jin H, Marler B, Guo H (2012). MCScanX: a toolkit for detection and evolutionary analysis of gene synteny and collinearity. Nucleic Acids Res.

[54] Chen C, Chen H, Zhang Y, Thomas HR, Frank MH, He Y, Xia R (2020). TBtools: an integrative toolkit developed for interactive analyses of big biological data. Mol Plant.

[55] Barros J, Escamilla-Trevino L, Song L, Rao X, Serrani-Yarce JC, Palacios MD, Engle N, Choudhury FK, Tschaplinski TJ, Venables BJ (2019). 4-Coumarate 3-hydroxylase in the lignin biosynthesis pathway is a cytosolic ascorbate peroxidase. Nat Commun.

