# Supplementary figures and images for: Genome-wide characterization of 2-oxoglutarate and Fe(II)-dependent dioxygenase family genes in tomato during growth cycle and their roles in metabolism
Source: BMC Genomics. 2021 Feb 18;22:126. doi: 10.1186/s12864-021-07434-3 (PMC7891033; doi:10.1186/s12864-021-07434-3)

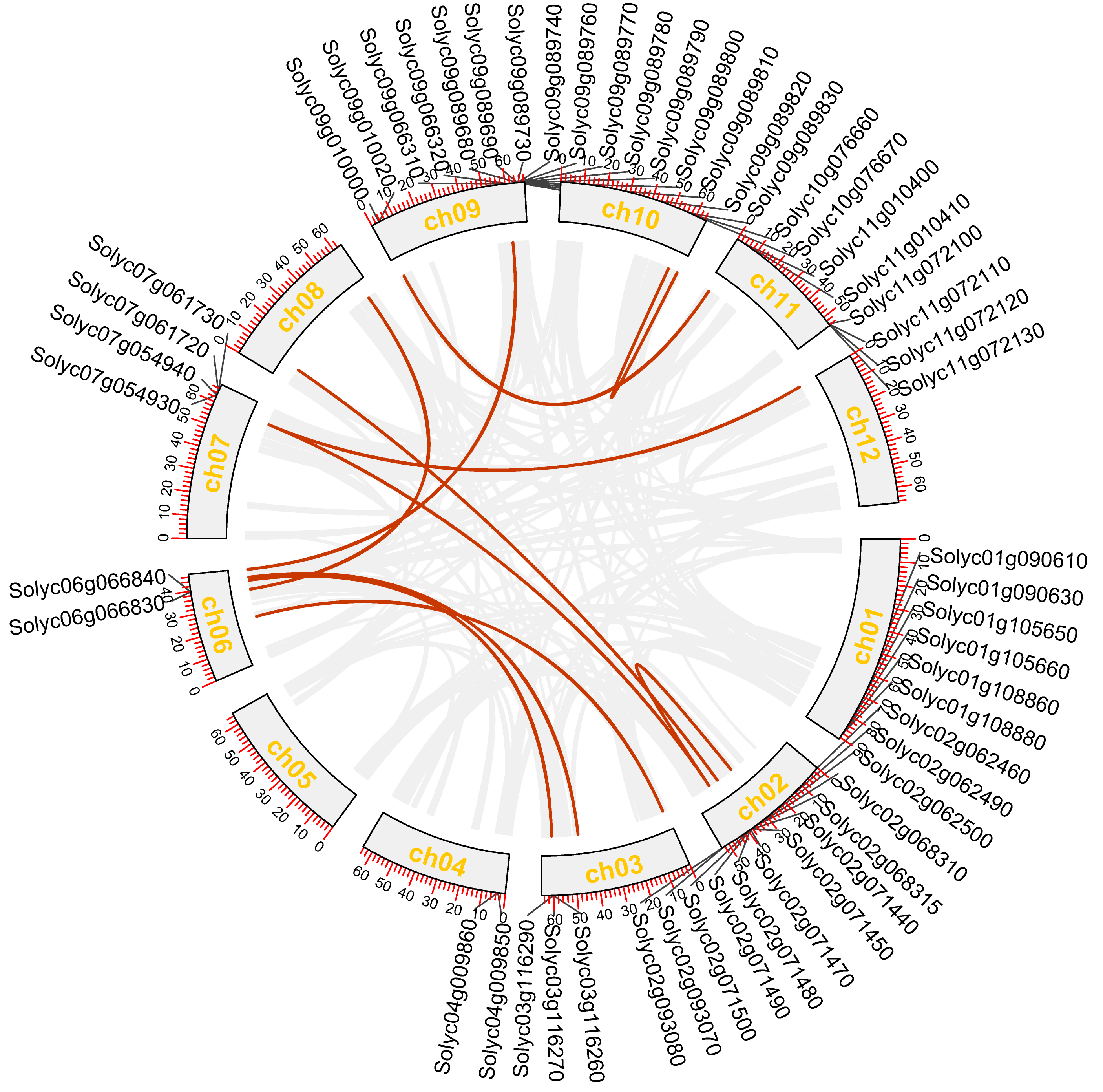

Supplement: Supplementary file 1 — Additional file 1: Fig. S1. Distributions of 54 tandem duplicated Sl2ODD genes in the tomato genome. The red lines indicated the duplicated Sl2ODD gene pairs (Fig. 3b). [file 12864_2021_7434_MOESM1_ESM.tif]
